# Impact of long-term antihypertensive and antidiabetic medications on the prognosis of post-surgical colorectal cancer: the Fujian prospective investigation of cancer (FIESTA) study

**DOI:** 10.18632/aging.101459

**Published:** 2018-05-24

**Authors:** Feng Peng, Dan Hu, Xiandong Lin, Binying Liang, Ying Chen, Hejun Zhang, Yan Xia, Jinxiu Lin, Xiongwei Zheng, Wenquan Niu

**Affiliations:** 1 Department of Cardiology, The First Affiliated Hospital of Fujian Medical University, Fuzhou, Fujian, China; 2 Department of Pathology, Fujian Cancer Hospital and Fujian Medical University Cancer Hospital, Fuzhou, Fujian, China; 3 Department of Medical Record, Fujian Cancer Hospital and Fujian Medical University Cancer Hospital, Fuzhou, Fujian, China; 4 Department of Core Research Laboratory, Fujian Cancer Hospital and Fujian Medical University Cancer Hospital, Fuzhou, Fujian, China; 5 Institute of Clinical Medical Sciences, China-Japan Friendship Hospital, Beijing, China; * Equal contribution

**Keywords:** colorectal cancer, diabetes mellitus, hypertension, medication, prognosis

## Abstract

Hypertension and diabetes mellitus are common comorbidities of colorectal cancer. We designed a prospective cohort study aiming to investigate the impact of long-term antihypertensive and antidiabetic medications on colorectal cancer-specific survival and recurrence among 713 post-surgical patients. All participants received radical resection for colorectal cancer during 2000-08, and they were followed up until July 2017. Colorectal cancer patients without hypertension had better survival than those with hypertension (median survival time [MST]: 190.3 months versus 99.0 months, *p* <0.001). The impact of antidiabetic medications on prolonging colorectal cancer survival was statistically significant, that is, patients receiving antidiabetic medications had longer survival time than untreated diabetic patients (MST: 135.8 months versus 80.2 months, *p*: 0.007), whereas the prognosis was greatly improved in colorectal cancer patients without diabetes mellitus (*p* <0.001). Medical treatment for hypertension and diabetes mellitus was associated with 28% (hazard ratio [HR]: 0.72; 95% confidence interval [CI]: 0.47-1.10; *p*: 0.131) and 57% (HR: 0.43; 95% CI: 0.22-0.82; *p*: 0.010) reduced risk of dying from colorectal cancer relative to those without medications, respectively. Our data indicate that long-term antidiabetic medications can significantly prolong the survival and improve the prognosis of post-surgical colorectal cancer.

## Introduction

Colorectal cancer is a leading cause of cancer-related death worldwide [1]. In China, colorectal cancer ranked fifth among the national death rates for both genders, causing approximate 191,000 million deaths in 2015 [2]. Colorectal cancer is frequently diagnosed at advanced stages, and survival rates largely depend on cancer stages at diagnosis [3]. According to the staging system defined by the American Joint Committee on Cancer (AJCC) 6^th^ edition system, the 5-year survival rate of colorectal cancer was 93.2% for stage I, 84.7% for stage IIa, 72.2% for stage IIb, 83.4% for stage IIIa, 64.1% for stage IIIb, 44.3% for stage IIIc and 8.1% for stage IV [4]. Because advanced patients often have poor prognosis, it is important to develop targeted prevention and early intervention strategies in control of colorectal cancer. From a clinical standpoint, surgery has long been established as the mainstay treatment for colorectal cancer [5]. The fact of matter, however, is that even after the surgery, colorectal cancer prognosis still is far from satisfying [6,7]. Consequently, current research interest has shifted to devise rational and effective strategies for improving the long-term prognosis of colorectal cancer.

Hypertension and diabetes mellitus are worldwide epidemics [8], and they are also regarded as common comorbidities of colorectal cancer [9-12]. A large cohort study by van Leersum et al. showed that hypertension and diabetes mellitus respectively affected about 22% and 11% of primary colorectal cancer patients [13]. Our recent findings revealed that pre-surgical hypertension and hyperglycemia were significant predictors for the poor prognosis of colorectal cancer after radical resection by prospectively inspecting the survival of 1,318 patients with median follow-up time of 58.6 months [14], which prompted us to speculate that long-term antihypertensive or antidiabetic medications can improve colorectal cancer survival. Indeed, some studies have suggested that the intake of antihypertensive and antidiabetic drugs was closely correlated with clinical outcomes of solid tumors at many sites, including colon and rectum [15,16]. For example, an analysis in 235 metastatic colorectal cancer patients indicated that the intake of beta-blockers in patients under chemotherapy played a potential prognostic role [17]. Moreover, a meta-analysis of seven cohorts demonstrated that metformin can prolong overall survival of diabetic patients with colorectal cancer, whereas the impact on colorectal cancer-specific survival remained non-significant [18]. However, the impact of antihypertensive and antidiabetic medications on post-surgical colorectal cancer survival is currently not fully understood.

In this prospective cohort study, we therefore aimed to investigate the impact of long-term antihypertensive and antidiabetic medications on colorectal cancer-specific survival and recurrence of post-surgical patients by analyzing data from the Fujian prospective investigation of cancer (FIESTA) study.

## RESULTS

### Baseline characteristics

In total, 713 participants with complete data on hypertension and diabetes mellitus, as well as medications were analyzed in this study, and baseline characteristics are shown in Table 1 and Table 2. There were 384, 246 and 83 colorectal cancer patients without hypertension, with untreated hypertension and with treated hypertension, respectively, and the corresponding numbers for diabetes mellitus were 385, 285 and 43. Patients without hypertension or diabetes mellitus were relatively younger and leaner (indexed by BMI) than those under treatment (*p* <0.001). Also, recurrence rate was significantly lower in patients without hypertension or diabetes mellitus than those under treatment (*p* <0.001), whereas the percentage of patients with distant metastasis was significantly higher in patients with untreated hypertension or diabetes mellitus (*p* <0.001). No significance was detectable for other characteristics across three groups for both hypertension and diabetes mellitus.

**Table 1:** Baseline characteristics of colorectal cancer patients per hypertension and medications.

**Characteristics**	**Patients w/o HT**	**Patients w/t untreated HT**	**Patients w/t treated HT**	***p***
Number	384	246	83	
Age, years	54.59 (12.74)	62.36 (11.77)	67.99 (8.85)	<0.001
Male gender	221 (57.55%)	135 (54.88%)	54 (65.06%)	0.268
Smoking	36 (9.38%)	32 (13.01%)	12 (14.46%)	0.226
Drinking	5 (1.30%)	8 (3.25%)	1 (1.20%)	0.198
Family history	27 (7.03%)	13 (5.28%)	3 (3.61%)	0.407
BMI, kg/m^2^	22.24 (3.03)	23.21 (3.17)	24.12 (2.99)	<0.001
Antihypertensive medications				
CCB	NA	NA	30 (36.14%)	
ACEI or ARB or beta-blocker	NA	NA	8 (9.64%)	
Other treatment	NA	NA	11 (13.25%)	
Unclear	NA	NA	34 (40.96%)	
Recurrence	17 (4.43%)	33 (13.41%)	10 (12.05%)	<0.001
Recurrence time (months)	14.1 (12.5, 14.1)	17.7 (12.3, 33.1)	21.95 (18.6, 43.5)	0.775
TNM stage				0.188
I/II	205 (53.39%)	128 (52.03%)	53 (63.86%)	
III/IV	179 (46.61%)	118 (47.97%)	30 (36.14%)	
Invasion depth				0.236
T1/T2	96 (25.00%)	55 (22.36%)	27 (32.53%)	
T3/T4	288 (75.00%)	191 (77.64%)	56 (67.47%)	
Regional LNM				0.056
N0	207 (53.91%)	138 (56.10%)	56 (67.47%)	
N1/N2	177 (46.09%)	108 (43.90%)	27 (32.53%)	
Distant metastasis	14 (3.65%)	30 (12.20%)	8 (9.64%)	<0.001
Differentiation				0.679
Low	279 (72.66%)	182 (73.98%)	57 (68.67%)	
Moderate/High	105 (27.34%)	64 (26.02%)	26 (31.33%)	
Tumor embolus	58 (15.10%)	44 (17.89%)	14 (16.87%)	0.495
Tumor size (cm)	5 (3.5, 6)	4.5 (3.5, 4.5)	4.5 (3.5, 6)	0.456
Number of LNM	0 (0, 2)	0 (0, 2)	0 (0, 1)	0.186

Abbreviations: w/t, with; w/o, without; HT, hypertension; BMI, body mass index; NA, not available; CCB, calcium channel blocker; ACEI, angiotensin converting enzyme inhibitor; ARB, angiotensin receptor blocker; TNM, tumor node metastasis; LNM, lymph node metastasis. Data are expressed as mean (standard deviation) or median (interquartile range) or count (percentage).

**Table 2:** Baseline characteristics of colorectal cancer patients per diabetes mellitus and medications.

**Characteristics**	**Patients w/o DM**	**Patients w/t untreated DM**	**Patients w/t treated DM**	***p***
Number	385	285	43	
Age, years	54.62 (12.74)	63.49 (11.58)	65.49 (9.86)	<0.001
Male	222 (57.66%)	165 (57.89%)	23 (53.49%)	0.858
Smoking	36 (9.35%)	39 (13.68%)	5 (11.63%)	0.213
Drinking	5 (1.30%)	8 (2.81%)	1 (2.33%)	0.374
Family history	27 (7.03%)	14 (4.91%)	2 (4.65%)	0.484
BMI, kg/m^2^	22.24 (3.03)	23.33 (3.20)	24.14 (2.71)	<0.001
Antidiabetic medications				
Metformin	NA	NA	5 (11.63%)	
Non-Metformin	NA	NA	21 (48.84%)	
Other treatment	NA	NA	9 (20.93%)	
Unclear	NA	NA	8 (18.60%)	
Recurrence	17 (4.42%)	38 (13.33%)	5 (11.63%)	<0.001
Recurrence time (months)	14.1 (12.5, 49.8)	19.4 (12.4, 33.1)	48.8 (8.3, 81.3)	0.102
TNM stage				0.502
I/II	205 (53.52%)	153 (53.68%)	27 (62.79%)	
III/IV	178 (46.48%)	132 (46.32%)	16 (37.21%)	
Invasion depth				0.665
T1/T2	97 (25.19%)	68 (23.86%)	13 (30.23%)	
T3/T4	288 (74.81%)	217 (76.14%)	30 (69.77%)	
Regional LNM				0.249
N0	208 (54.03%)	166 (58.25%)	28 (65.12%)	
N1/N2	177 (45.97%)	119 (41.75%)	15 (34.88%)	
Distant metastasis	14 (3.64%)	36 (12.63%)	2 (4.65%)	<0.001
Differentiation				0.088
Low	279 (72.47%)	199 (87.37%)	40 (93.02%)	
Moderate/High	106 (27.53%)	36 (12.63%)	3 (6.98%)	
Tumor embolus	58 (15.06%)	54 (18.95%)	4 (9.30%)	0.038
Tumor size (cm)	5 (3.5, 6)	4.5 (3.5, 6)	4 (3, 6)	0.517
Number of LNM	0 (0, 2)	0 (0, 2)	0 (0, 1)	0.337

Abbreviations: w/t, with; w/o, without; DM, diabetes mellitus; BMI, body mass index; NA, not available; TNM, tumor-node-metastasis; LNM, lymph node metastasis. Data are expressed as mean (standard deviation) or median (interquartile range) or count (percentage) if appropriate.

### Overall survival comparison

**Figure 1:**
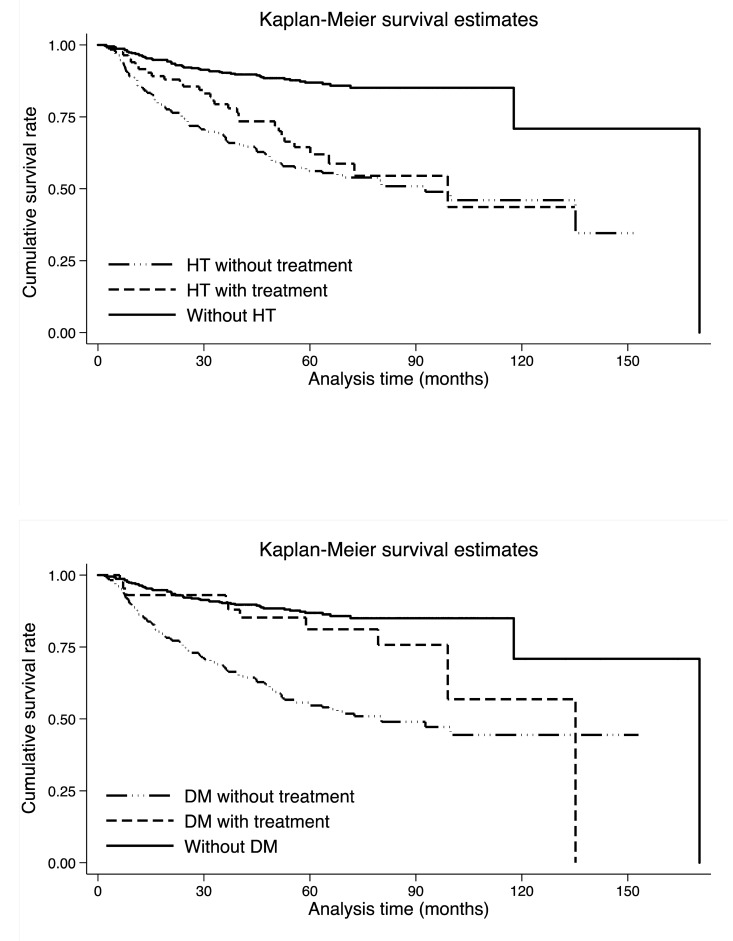
**Kaplan-Meier survival curves per hypertension (the upper panel) and diabetes mellitus (the lower panel).** Abbreviations: HT, hypertension; DM, diabetes mellitus. There were 384, 246 and 83 colorectal cancer patients without hypertension, with untreated hypertension and with treated hypertension, respectively. There were 385, 285 and 43 colorectal cancer patients without diabetes mellitus, with untreated diabetes mellitus and with treated diabetes mellitus, respectively. The Log-rank test was statistically significant for both hypertension and diabetes mellitus medications (*p* <0.001).

Figure 1 shows the comparison of cumulative survival rates per hypertension and diabetes mellitus in colorectal cancer patients. As expected, colorectal cancer patients without hypertension had better survival than those with hypertension (median survival time (MST): 190.3 months versus 99.0 months, Log-rank test *p* <0.001 in Supplementary Table 1). Although antihypertensive medications seemed to be beneficial on colorectal cancer prognosis, the impact was not statistically significant (Log-rank test *p*: 0.205). By contrast, in colorectal cancer patients with diabetes mellitus, the impact of medications on survival was statistically significant, that is, patients receiving antidiabetic medications had longer survival time than untreated diabetic patients (MST: 135.8 months versus 80.2 months, Log-rank test *p*: 0.007), whereas the prognosis was greatly improved in colorectal cancer patients without diabetes mellitus (MST: 170.3 months, Log-rank test *p* <0.001 in Supplementary Table 1).

Combined impact of hypertension and diabetes mellitus medications on colorectal cancer survival is presented in Supplementary Figure 1.

### Overall recurrence comparison

**Figure 2:**
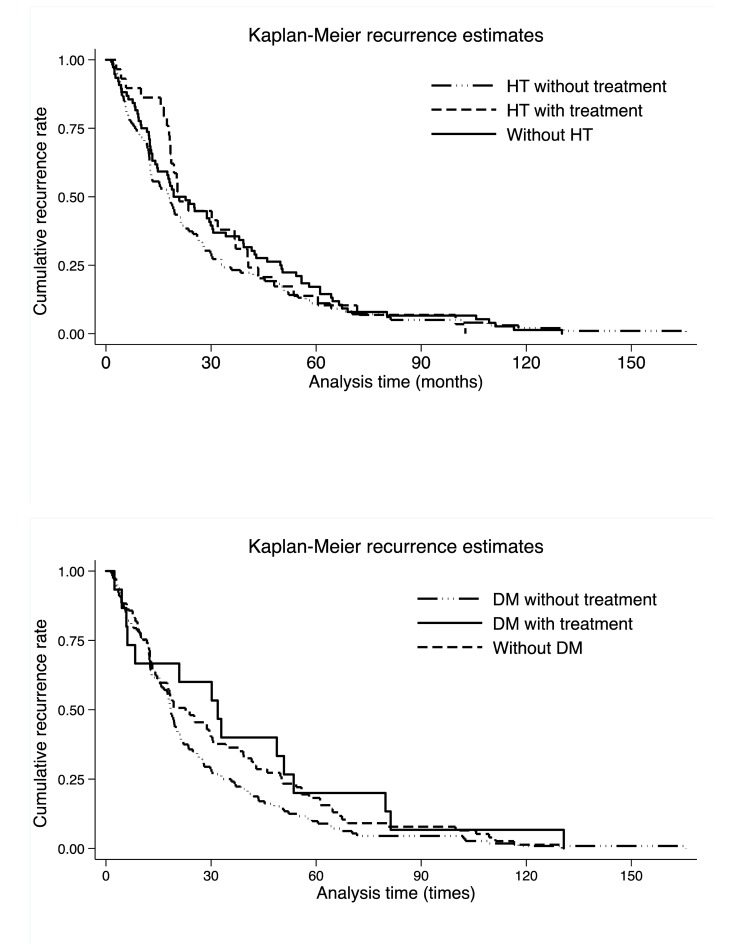
**Kaplan-Meier recurrence curves per hypertension (the upper panel) and diabetes mellitus (the lower panel).** Abbreviations: HT, hypertension; DM, diabetes mellitus. There were 33, 10 and 17 colorectal cancer patients without hypertension, with untreated hypertension and with treated hypertension, respectively. There were 17, 38 and 5 colorectal cancer patients without diabetes mellitus, with untreated diabetes mellitus and with treated diabetes mellitus, respectively. Log-rank test was not significant for both hypertension and diabetes mellitus medications (*p*: 0.899 for hypertension and 0.169 for diabetes mellitus).

The comparison of cumulative recurrence rates per hypertension and diabetes mellitus in colorectal cancer patients is provided in Figure 2, and no significance was observed across hypertension or diabetes mellitus, as well as across medications (Log-rank test *p*: 0.899 for hypertension and 0.169 for diabetes mellitus).

### Survival comparison per medications

**Figure 3:**
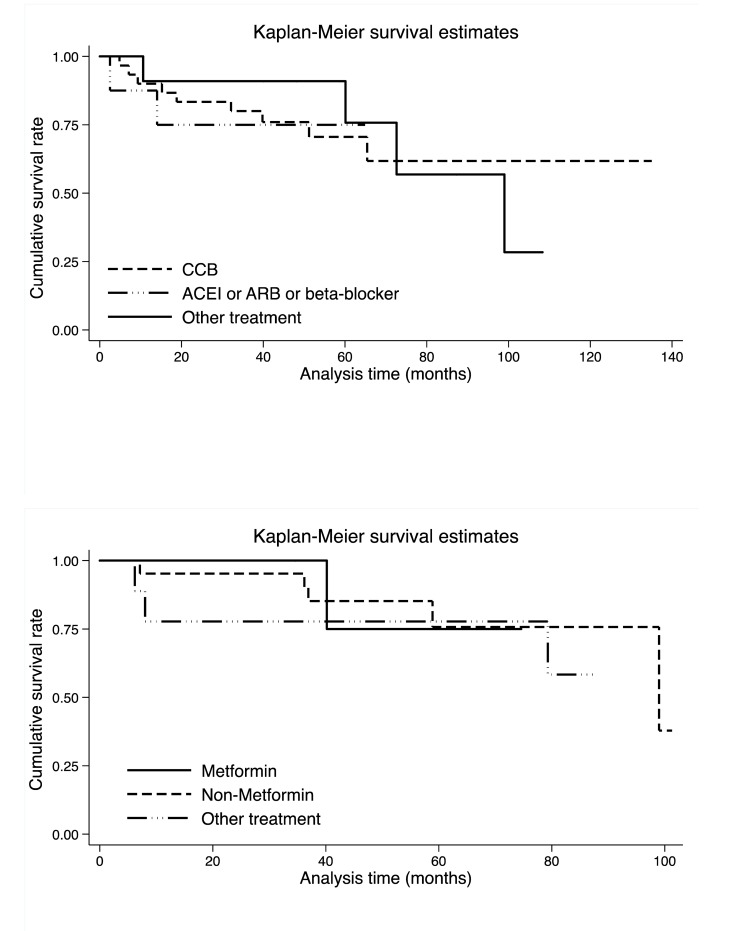
**Kaplan-Meier survival curves per antihypertensive (the upper panel) and antidiabetic (the lower panel) medications.** Abbreviations: CCB, calcium channel blocker; ACEI, angiotensin converting enzyme inhibitor; ARB, angiotensin receptor blocker. There were 30, 8 and 11 colorectal cancer patients who took ACEI or ARB or beta-blocker, CCB and other antihypertensive drugs, respectively. There were 5, 21 and 9 colorectal cancer patients who took metformin, non-metformin and other antidiabetic drugs, respectively. Log-rank test was not significant for both antihypertensive (*p*: 0.978) and antidiabetic (*p*: 0.778) medications.

Shown in Figure 3 is the comparison of cumulative survival rates per antihypertensive and antidiabetic medications in colorectal cancer patients. Upon stratification by antihypertensive medications, colorectal cancer patients with other medications seemed to have better survival within 60 months after the surgery than those receiving CCBs and ACEIs or ARBs or beta-blockers, whereas there was no detectable significance (Log-rank test *p*: 0.978 in Supplementary Table 1). By contrast, upon stratification by antidiabetic medications, colorectal cancer patients with metformin only seemed to have better survival within 40 months after the surgery than patients with non-metformin and the other medications, and similarly no significance was noted (Log-rank test *p*: 0.778 in Supplementary Table 1).

### Mortality risk estimation

To see whether antihypertensive or antidiabetic medications can improve the prognosis of post-surgical colorectal cancer, we first tested whether proportional-hazards assumption was satisfied. As shown in Supplementary Figure 2, this assumption was violated for the comparison of colorectal cancer patients with and without antihypertensive or antidiabetic medications because the two curves were not parallel. As the ln(-ln(*S(t)*)) is a linear function of ln(*t*) (here, *t* is the time variable and *S(t)* is the survival function), we therefore adopted the multivariate Weibull proportional hazards regression model to derive survival estimates.

Table 3 presents the overall and stratified estimates for colorectal cancer mortality risk per hypertension and diabetes mellitus, respectively. All risk estimates were adjusted for age, gender, BMI, smoking, drinking and family history of cancer. Overall, medical treatment for hypertension and diabetes mellitus was associated with 28% (HR: 0.72; 95% CI: 0.47-1.10; *p*: 0.131) and 57% (HR: 0.43; 95% CI: 0.22-0.82; *p*: 0.010) reduced risk of dying from colorectal cancer relative to those without medications, respectively, especially for diabetes mellitus. In case of diabetes mellitus, the power to detect this significant association was over 99.0%.

**Table 3:** Overall and stratified analyses of antihypertensive and antidiabetic medications in predicting the mortality risk of post-surgical colorectal cancer.

**Groups**	**Antihypertensive medications**					**Antidiabetic medications**			
	**Deaths/Patients**	**HR**	**95% CI**	***p***		**Deaths/Patients**	**HR**	**95% CI**	***p***
Overall	140/327	0.72	0.47-1.10	0.131		140/327	0.43	0.22-0.82	0.010
Gender									
Males	85/187	0.72	0.42-1.21	0.213		85/187	0.54	0.25-1.18	0.125
Females	55/140	0.71	0.34-1.47	0.356		55/140	0.30	0.01-0.97	0.045
TNM stage									
I/II	45/180	0.81	0.41-1.60	0.544		45/180	0.68	0.26-1.74	0.415
III/IV	95/147	0.86	0.49-1.51	0.606		95/147	0.30	0.12-0.73	0.009
Invasion depth									
T1/T2	21/81	1.39	0.54-3.58	0.495		21/81	0.38	0.09-1.65	0.196
T3/T4	119/246	0.65	0.40-1.07	0.090		119/246	0.46	0.22-0.95	0.037
Regional LNM									
N0	58/194	0.79	0.43-1.45	0.455		58/194	0.64	0.27-1.51	0.313
N1/N2	39/71	1.14	0.50-2.57	0.757		39/71	0.40	0.14-1.12	0.081
Distant metastasis									
Negative	104/209	0.74	0.46-1.20	0.222		104/290	0.44	0.21-0.91	0.027
Positive	36/37	0.59	0.21-1.68	0.325		36/37	1.86	0.34-10.15	0.476
Tumor differentiation									
Low	93/238	0.52	0.29-0.93	0.028		93/238	0.39	0.19-0.80	0.011
Moderate/High	22/36	0.44	0.14-1.35	0.153		22/36	6.40	0.72-56.63	0.095
Tumor embolus									
Positive	38/57	0.32	0.11-0.95	0.040		38/57	0.16	0.02-1.18	0.072
Negative	55/171	0.79	0.43-1.48	0.468		55/171	0.60	0.28-1.27	0.182
Tumor size									
≤ 4.5 cm	64/170	0.92	0.51-1.66	0.782		64/170	0.59	0.26-1.31	0.193
> 4.5 cm	75/155	0.52	0.28-0.98	0.043		75/155	0.30	0.09-0.96	0.042

Abbreviations: HR, hazard ratio; 95% CI, 95% confidence interval; TNM, tumor-node-metastasis; LNM, lymph node metastasis. Besides stratification upon gender, p value was adjusted for age, gender, body mass index, smoking, drinking and family history of cancer.

By gender, TNM stage and invasion depth, the reduced risk was only significant when analysis was restricted to antidiabetic medications (HR: 0.30, 0.30 and 0.46; 95% CI: 0.01-0.97, 0.12-0.73 and 0.22-0.95; *p*: 0.045, 0.009 and 0.037, respectively) (Table 3). By distant metastasis, antidiabetic medications had a significantly reduced risk only in patients without distant metastasis (HR: 0.44; 95% CI: 0.21-0.91; *p*: 0.027). By tumor differentiation, both antihypertensive and antidiabetic medications can significantly reduce the risk of colorectal cancer-specific mortality (HR: 0.52 and 0.39; 95% CI: 0.29-0.93 and 0.19-0.80; *p*: 0.028 and 0.011, respectively) in patients with low differentiation. By tumor embolus, risk reduction was only seen in positive embolus for the comparison of patients with antihypertensive medications than those without (HR: 0.32; 95% CI: 0.11-0.95; *p*: 0.040). By tumor size, both antihypertensive and antidiabetic medications can reduce the mortality risk of colorectal cancer significantly in patients with tumor size greater than 4.5 cm (HR: 0.52 and 0.30; 95% CI: 0.28-0.98 and 0.09-0.96; *p*: 0.043 and 0.042, respectively).

## DISCUSSION

As an extension of our previous FIESTA study [14,19-26], we investigated the impact of long-term antihypertensive and antidiabetic medications on colorectal cancer-specific survival and recurrence in post-surgical patients. Noteworthily, our data indicate that long-term antidiabetic medications can significantly prolong the survival and improve the prognosis of post-surgical colorectal cancer, whereas the anticancer impact of long-term antihypertensive medications was not obvious. Our findings highlight the importance of enhanced screening and targeted management of diabetes mellitus in colorectal cancer patients, especially after radical resection.

The association of diabetes mellitus with the risk and prognosis of colorectal cancer has been widely evaluated in the literature [27-29]. Mills et al. conducted a meta-analysis of 26 studies and found that colorectal cancer patients complicated with diabetes mellitus were at 17% and 12% significantly increased risk of all-cause and cancer-speciﬁc mortality, respectively [30]. In this present study, we found that diabetic patients had worse cancer-specific prognosis of post-surgical colorectal cancer than patients without diabetes mellitus, yet failed to detect any significance in association with recurrence, possibly due to small sample size involved. Our findings further revealed that long-term antidiabetic medications can prolong the survival and reduce colorectal cancer-specific mortality risk by about 50%, whereas there was no distinction in prognosis between metformin, a widely used oral antidiabetic agent, and non-metformin medications. In support of our findings, a recent meta-analysis of 7 cohort studies conducted by Meng et al. indicated that relative to non-metformin, metformin had no benefits for cancer-specific survival, whereas metformin can remarkably prolong the overall survival of colorectal cancer patients with comorbid diabetes mellitus [18]. There is mounting evidence underscoring the important anticancer impact of metformin on carcinogenesis, likely through the upregulation of AMP-activated protein kinase (AMPK) activity and the downstream suppression of signaling through the mTOR [31,32]. In addition, animal studies provided evidence that metformin can inhibit both colon carcinoma and intestinal polyp growth [33,34]. Although epidemiologic evidence failed to support the beneficial superiority of metformin over non-metformin, we cannot completely negate its existence, as diabetic patients may take more than one medication to lower blood glucose at the same time. In fact, during the follow-up of this study, we found that patients receiving either metformin or non-metformin usually took other types of antiglycemic drugs to better control glucose. Even in clinical settings, it is impractical and unethical to assign study participants to mono-medical treatment. It is also evidenced that metformin was associated with an increased risk of colorectal cancer in males, but not in females. In view of small sample sizes in the present study, we cannot interrogate the gender-specific difference between metformin and non-metformin in colorectal cancer survival [35]. We agree that future large prospective studies are needed to see whether gender plays a role in predicting the risk and prognosis of colorectal cancer.

By contrast, our results did not explicitly support a favorable benefit profile of antihypertensive medications for the prognosis of post-surgical colorectal cancer, and distinction in prognosis between antihypertensive drugs was nonsignificant. Epidemiological evidence suggests a potential prognostic role of beta-blockers in colorectal cancer patients under chemotherapy. Moreover, two meta-analyses consistently demonstrated that bevacizumab-induced hypertension may represent a prognostic factor in patients with metastatic colorectal cancer [36,37]. However, no report has thus far addressed the relationship between antihypertensive medications and post-surgical colorectal cancer prognosis. So, whether improvement in colorectal cancer survival with antihypertensive medications is because of only blood pressure lowering or additional anticancer mechanisms is still an open question, and further validation of our findings will be helpful.

Finally, several limitations should be acknowledged. Firstly, this study was carried out in a mono-center, and our findings could better be generalized pending consistently validated in other cohorts. However, mono-center design may represent an important indicator of daily clinical practice. Secondly, all colorectal cancer patients were enrolled between January 2000 and December 2008, and during the 9-year period remarkable advances in surgical techniques might introduce a possible bias, which may underestimate the impact of antihypertensive or antidiabetic medications during follow-up on colorectal cancer mortality. Thirdly, all participants were followed up at the Outpatient Department of Fujian Cancer Hospital or through calling or letters to obtain information on antihypertensive and antidiabetic medications, whereas their blood pressure and glucose were not measured. In case of poorly controlled blood pressure or glucose, participants were recommended to comprehensive or specialized hospitals. Fourthly, the findings presented in this study are based on only colorectal cancer patients who are operable for radical resection and therefore cannot be directly extrapolated to the general population. For this reason, validation in other large cohorts is required.

Taken together, our data indicate that long-term antidiabetic medications can significantly prolong the survival and improve the prognosis of post-surgical colorectal cancer, whereas the anticancer impact of long-term antihypertensive medications was not obvious. This study underscores the critical need for intensified screening programs and pharmacological management for diabetes mellitus in colorectal cancer patients to prolong the survival and improve the prognosis in China.

## MATERIALS AND METHODS

### The FIESTA study

The FIESTA study is an ongoing prospective evaluation on the survival of post-surgical patients with common digestive tract cancer (such as esophageal cancer, gastric cancer and colorectal cancer) initiated in January 2000 and still recruiting [14,19-26]. Approval of this study was obtained from the Ethics Committees of Fujian Cancer Hospital. All participants gave written informed consent before recruitment.

### Study participants

All participants were consecutively recruited from the Department of Thoracic Surgery at Fujian Cancer Hospital during the period between January 2000 and December 2008, and they were followed up until July 2017. Only participants of Han Chinese descent and without consanguinity were eligible for inclusion, and they for the first time underwent radical resection for colorectal cancer that was confirmed by pre-surgical biopsy or post-surgical pathologic tests. In addition, no participants had received chemotherapy and radiotherapy. Only participants with complete data on hypertension or diabetes mellitus and their medications were analyzed. Moreover, they must be followed up for one month or over.

### Tissue collection

From each participant, both cancerous tissue and normal colonic or rectal tissue were resected during the surgery. Tissue samples were formalin-fixed and paraffin-embedded, and their pathological characteristics were analyzed at the Department of Pathology, Fujian Cancer Hospital.

### Follow-up evaluation

After discharge from hospital, annual follow-up was carried out for qualified participants at the Out-Patient Department of Fujian Cancer Hospital or through calling or sending printed letters in case of no-show on scheduled time. In case of death events, the date was recorded from relatives or medical reports. The primary outcome was death from colorectal cancer. Survival time was calculated from the date of receiving radical resection to the date of death or last follow-up, whichever occurred first. Median follow-up time was also calculated, and it referred to the length of time from the start of radical resection for colorectal cancer to the time when half of the patients were still alive.

### Baseline characteristics

A self-designed questionnaire was completed by each participant at the time of admission on age, gender, smoking, drinking, family cancer history, antihypertensive medications and antidiabetic medications. Meanwhile, body weight and height were measured with participants wearing light clothing and no shoes, and body mass index (BMI) was calculated as weight divided by height in meters-squared (kg/m^2^). Arterial blood pressure was measured with participants in the seated position using a mercury sphygmomanometer, and three readings were taken, each at least 5 minutes apart. Age was recorded at the date of receiving radical resection for colorectal cancer. Cigarette smoking was defined by indicator variables for never, former and current smoking. Alcohol drinking was defined by indicator variables for never, former and current drinking. Family cancer history was recorded for study participants with one or more affected relatives within three generations who suffered malignancies except non-melanoma skin cancer.

Hypertension and diabetes mellitus, as well as information on medications were recorded during the annual follow-up period. Hypertension was diagnosed if averaged blood pressure was at least 140 mm Hg systolic or 90 mm Hg diastolic, or when the participants were on antihypertensive medications. In view of sample sizes involved, the type of antihypertensive medications was classified into the calcium channel blocker (CCB) group, the angiotensin converting enzyme inhibitor (ACEI) or angiotensin receptor blocker (ARB) or beta-blocker group and the other treatment group (including irregular treatment and use of traditional Chinese medicine). Fasting venous blood samples were taken the morning of surgery day and used to measure blood glucose using an automated glucose oxidase method at Clinical Laboratory, Fujian Cancer Hospital. Diabetes mellitus was diagnosed if the fasting blood glucose ≥7.0 mmol/L or when the participants were on antidiabetic medications. In view of sample sizes involved, the type of antidiabetic medications was classified into the metformin group, the non-metformin group and the other treatment group (including irregular treatment and use of traditional Chinese medicine).

Clinicopathologic characteristics including tumor-node-metastasis (TNM) stage (I, II, III and IV) [38], depth of invasion (T1, T2, T3 and T4), regional lymph node metastasis (N0, N1, N2 and N3), distant metastasis (M0 and M1), tumor differentiation, tumor embolus and tumor size (in centimeters) were obtained from pathological reports.

### Statistical analysis

Baseline characteristics were expressed as mean (standard deviation) or median (interquartile range) or count (percentage) if appropriate. Kaplan-Meier curves and Log-rank tests were used to present and test the differences in cumulative survival or recurrence rates. Tests of proportional-hazards assumption were performed to facilitate the selection of proper model. The risk prediction of pre-surgical hypertension or diabetes mellitus and their medications for colorectal cancer mortality was calculated under the Cox or Weibull, where appropriate, proportional hazards regression model, and the magnitude of risk prediction was denoted by hazard ratio (HR) and 95% confidence interval (95% CI).

All statistical analyses were done by the STATA/SE Release 14.0 (StataCorp, College Station, TX, USA). Study power was estimated by the PS Power and Sample Size Calculations software Release 3.0 [39].

## Supplementary Material

Supplementary File
