# Supplementary material for: Impact of long-term antihypertensive and antidiabetic medications on the prognosis of post-surgical colorectal cancer: the Fujian prospective investigation of cancer (FIESTA) study
Source: Aging (Albany NY). 2018 May 24;10(5):1166–81. doi: 10.18632/aging.101459 (PMC5990395; doi:10.18632/aging.101459)
Supplement: Supplementary File [file aging-10-101459-s001.pdf]

SUPPLEMENTARY MATERIAL

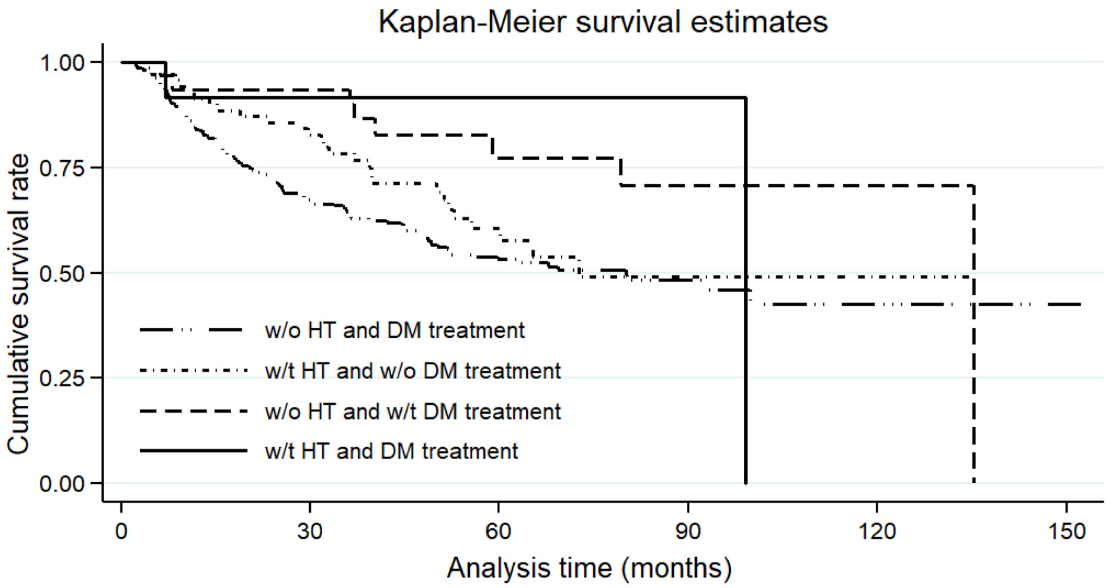

Supplementary Figure S1. Combined impact of hypertension and diabetes mellitus medications on colorectal cancer survival. Abbreviations: HT, hypertension; DM, diabetes mellitus; w/t, with; w/o, without.

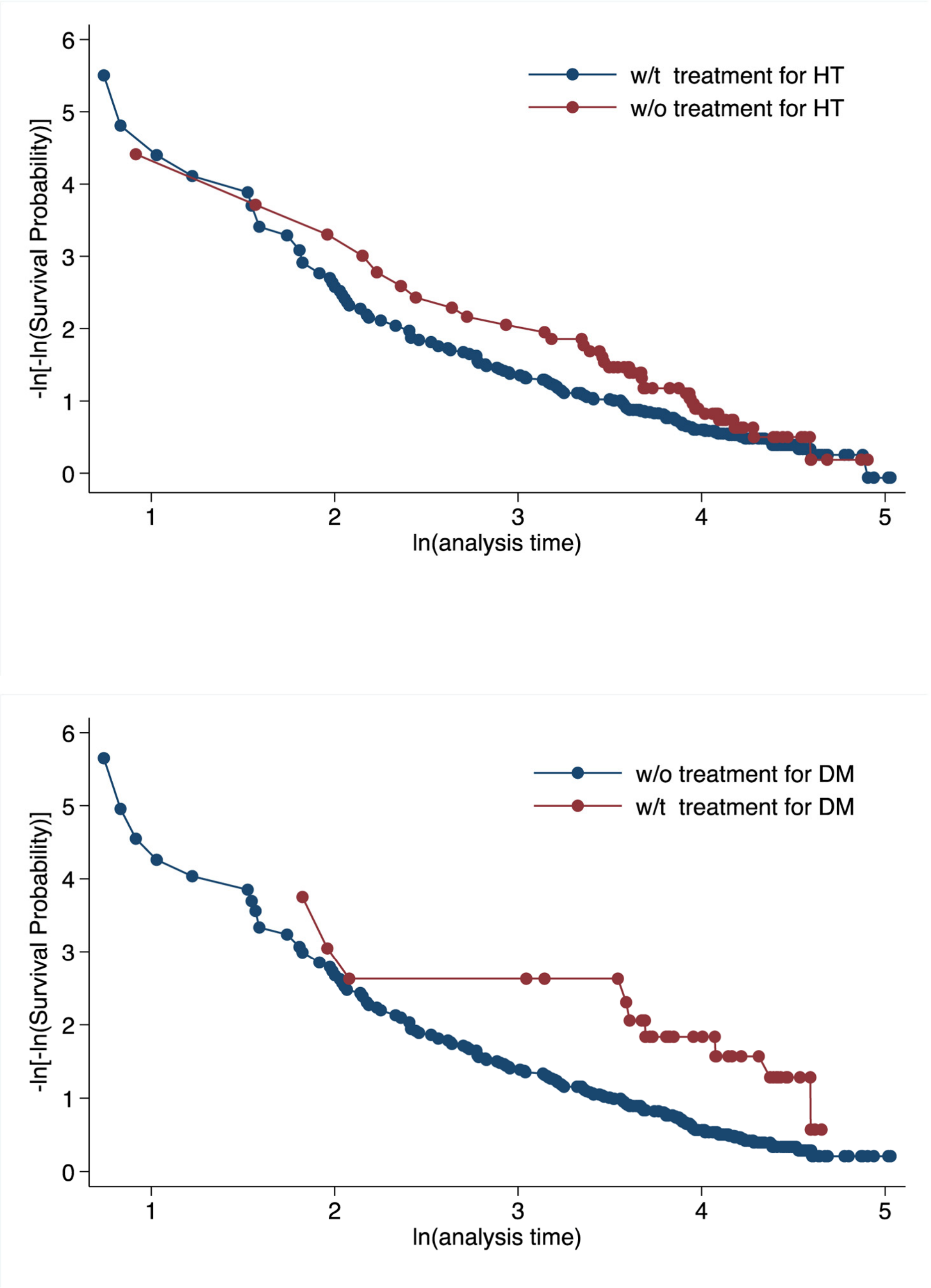

Supplementary Figure S2. Testsof proportional-hazards assumption for antihypertensive (the upper panel) and antidiabetic (the lower panel) medications. Abbreviations: HT, hypertension; DM, diabetes mellitus; w/t, with; w/o, without.

Supplementary Table 1. Summaries of Kaplan-Meier survival estiamtes per hypertension and diabetes mellitus status, as well as their specific edications.

Medications	Time at risk	Survival rate at quantiles			Log-rank test	*p*
		25%	50%	75%	Chi-squared	
Hypertension (HT)						
Patients without HT	23498.4	117.7	170.3	170.3	81.94	<0.001
Patients with untreated HT	12542.7	24.0	92.7	-*		
Patients with treated HT	4240.7	39.8	99.0	-		
Antihypertensive medications						
CCB	1585.8	51.2	-	-	0.04	0.978
ACEI or ARB or beta-blocker	326.8	14.0	-	-		
Other treatment	706.5	72.6	99.0	-		
Diabetes mellitus (DM)						
Patients without DM	23522.5	117.7	170.3	170.3	89.2	<0.001
Patients with untreated DM	14165.3	24.8	80.2	-		
Patients with treated DM	2594.0	99.0	135.2	135.2		
Antidiabetic medications						
Metformin	281.6	40.2	-	-	0.5	0.778
Non-Metformin	1205.1	99.0	99.0	-		
Other treatment	497.0	79.3	-	-		

Abbreviations: CCB, calcium channel blocker; ACEI, angiotensin converting enzyme inhibitor; ARB, angiotensin receptor blocker. *Data are not available.
